# Patient satisfaction and side effects in primary care: An observational study comparing homeopathy and conventional medicine

**DOI:** 10.1186/1472-6882-8-52

**Published:** 2008-09-18

**Authors:** Florica Marian, Kerstin Joost, Krishan D Saini, Klaus von Ammon, André Thurneysen, André Busato

**Affiliations:** 1 Institute for Complementary Medicine KIKOM, University of Bern, Imhoof Pavillon, Inselspital, CH-3010, Bern, Switzerland; 2 Institute for Evaluative Research in Orthopaedic Surgery, University of Bern, Stauffacherstrasse 78, CH-3014, Bern, Switzerland

## Abstract

**Background:**

This study is part of a nationwide evaluation of complementary medicine in Switzerland (Programme Evaluation of Complementary Medicine PEK) and was funded by the Swiss Federal Office of Public Health. The main objective of this study is to investigate patient satisfaction and perception of side effects in homeopathy compared with conventional care in a primary care setting.

**Methods:**

We examined data from two cross-sectional studies conducted in 2002–2003. The first study was a physician questionnaire assessing structural characteristics of practices. The second study was conducted on four given days during a 12-month period in 2002/2003 using a physician and patient questionnaire at consultation and a patient questionnaire mailed to the patient one month later (including Europep questionnaire).

The participating physicians were all trained and licensed in conventional medicine. An additional qualification was required for medical doctors providing homeopathy (membership in the Swiss association of homeopathic physicians SVHA).

**Results:**

A total of 6778 adult patients received the questionnaire and 3126 responded (46.1%). Statistically significant differences were found with respect to health status (higher percentage of chronic and severe conditions in the homeopathic group), perception of side effects (higher percentage of reported side effects in the conventional group) and patient satisfaction (higher percentage of satisfied patients in the homeopathic group).

**Conclusion:**

Overall patient satisfaction was significantly higher in homeopathic than in conventional care. Homeopathic treatments were perceived as a low-risk therapy with two to three times fewer side effects than conventional care

## Background

Homeopathy is one of the most practiced complementary therapies in Switzerland and Europe [1-4]. Despite the fact that the effectiveness of homeopathy is still subject to controversial discussions [5-8], investigations show that patient satisfaction is high [3,9]. Important reasons for patients to consult a homeopathic physician (HP) are related to limited effectiveness of conventional medicine in cases of chronic diseases, adverse side-effects of drugs, and the invasiveness of conventional medicine [10-13]. Also, the quality of the physician-patient relationship seems to be a key factor [14,15].

Since the 1980s, patient satisfaction has been recognized as an important factor in the assessment of the quality of health services [16,17]. To date, there has been no survey comparing patient outcomes in homeopathy and conventional care in Switzerland. [18]. Therefore, we examined patient satisfaction and perceptions of side effects for homeopathic treatment deriving from an observational study conducted between 2002 and 2003. The present study was part of the Complementary Medicine Evaluation Project (PEK), aimed at the evaluation of five complementary therapies (homeopathy, anthroposophic medicine, herbal therapy, neural therapy and traditional Chinese medicine) in Switzerland. The project was funded by the Swiss Federal Office of Public Health [19].

## Methods

### Physicians and patients

**Figure 1:**
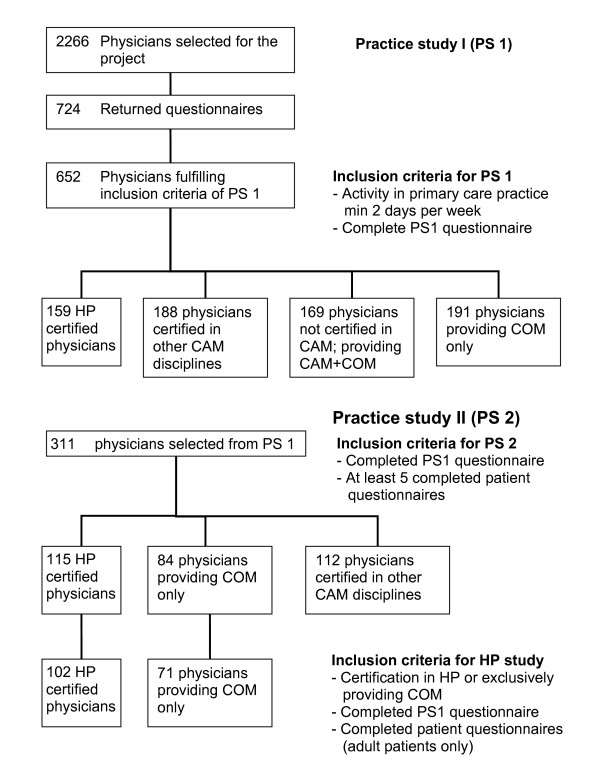
Flowchart of sampling procedures.

Eligibility criteria for all participating physicians were training and license in conventional medicine, and medical activity in primary care for at least two days per week (Figure 1). An additional qualification in homeopathy recognized by the Swiss Medical Association (FMH) was required of medical doctors providing homeopathy.

All members of the Swiss Association of Homeopathic Physicians (SVHA) in 2002 (n = 282) were asked to take part in the PEK study and are referred to as HP (homeopathic physicians. A random sample of physicians working as primary care providers and not listed in any medical society for complementary and alternative medicine, referred to here as CP (conventional physicians), was also recruited. This sample was compiled from the complete membership list of the FMH and was proportionally matched to the regional distribution of physicians providing homeopathy. Both membership lists of the SVHA and FMH, represent complete census data of all respective physicians providing ambulatory care in Switzerland.

Inclusion criteria for patients were written informed consent, ability to read and write German, French, or Italian, and age above 16 years. Given these physician and patient based eligibility criteria 71 CP and 102 HP were finally included in the study (Figure 1).

### Data collection

Sampling was performed in two parts. The first part (Practice study I) was aimed at structural attributes of physicians and practices, and the second part at characteristics of processes of care (Practice study II), including a physician- and patient-based documentation of consultations and outcomes (Figure 1). Data collection was conducted between 2002 and 2003. Patient satisfaction data were obtained from the second part and were associated with structural aspects of care obtained in the first part. The study design was purely observational, without interference into treatment choices of physicians and patients. Physicians and their staff were instructed to sample consecutive patients consulting their practice on four given days during a 12 month period. Days on which data were sampled were defined by the study coordinator and equally distributed across weekdays. Sampling of data related to the processes of care was also performed in two steps: prior to the consultation volunteering patients were asked about their health status and demographic aspects. Physicians documented the subsequent consultation with reference to diagnosis, duration of problems, comorbidities, and diagnostic and therapeutic procedures. It is important to mention that practitioners specializing in homeopathy were free to use homeopathy, conventional medicine, or any other treatment. Nevertheless, all patients treated by members of the association SVHA were allocated to the group of "homeopathic patients." Four weeks after the initial consultation, patients received a postal questionnaire collecting data about their health status, perceived treatment effects, frequency of side effects, satisfaction with the treatment, and fulfillment of their treatment-related expectations. A second part of the questionnaire was aimed at patient satisfaction in particular, and a Europep questionnaire (European Task Force on Patient Evaluation of Practice) was included [20]. This questionnaire has 23 questions, each with a five-point answer scale ranging from poor to excellent, dealing with 5 main dimensions: relations and communications, medical care, information and support, continuity and cooperation, facilities availability and accessibility.

Data collection procedures were developed in close cooperation with an interdisciplinary group that included experts in conventional and complementary medicine. All patients and physicians participated on a voluntary basis, and the physicians received 500 Swiss Francs (300 Euros) as compensation for their time. The ethics committee of the Canton Bern raised no objection to the study.

### Data management and data analysis

All data were recorded using a relational database. Physicians' free-text answers regarding main and secondary diagnoses were coded according to the ICD-10 classification by two physicians and a pharmacist. In case of uncertainty, classification was achieved after reaching consensus within the research group. Patients with a disease duration of more than three months were defined as chronic and the remainder as acute, according to the definition of the U.S. National Center for Health Statistics. Data analysis was performed in two steps. A first step included descriptive analyses using tables and graphs. In a second step, continuous target variables were analyzed with multivariate linear models and adjusted means were given as least square means (LS-means). Ordinal outcomes were reduced to two level scales with the most favourable answer category coded as one and all other non-missing categories as zero. These data were analyzed using multivariate logistic regression models. Covariables of multivariate models were defined a priori and were used to adjust for demographic factors of patients (age, gender and educational status) and for chronicity of health problems (coded as 0 for < 3 months and 1 for ≥ 3 months). All analytical procedures accounted for clustering of observations at the practice level using Taylor series expansion procedures for the 2*2 tables and mixed effects models for multivariate procedures [21]. 95% confidence intervals (95% CI) of means, proportions and odds ratios were calculated accordingly. The level of significance was set at p < 0.05 throughout the study and SAS 9.1 (SAS Institute Inc., Cary, NC, USA) was used for all calculations.

## Results

### Characteristics of physicians and practices (Table 1)

**Table 1:** Structural characteristics of physicians and practices

		**CP**		**HP**	
		**#**	**%**	**#**	**%**
**Physicians**	N	71	41.8	99	58.2

Gender*	Male/female	62/9	87.3/12.7	68/31	68.7/31.3

**Years since graduation**	Mean	23.44			21.85
Language*	German	43	60.6	78	78.8
	French	25	35.2	18	18.2
	Italian	3	4.2	3	3.0

Urbanisation*	City	24	33.8	56	56.6
	Suburb	35	49.3	33	33.3
	Rural area	12	16.9	10	10.1

Practice type*	Single	51	71.8	51	51.5
	Group	20	28.2	48	48.5

**Practice equipment**	Laboratory*	68	95.8	66	66.7
	ECG*	69	97.2	66	66.7
	X-ray*	57	80.3	19	19.2
	Ultrasound	16	22.5	7	7.1

Professional activity*	fulltime	64	91.4	62	66.0
	parttime	6	8.6	32	34.0

Consultation time (min.)*	mean		16.9		28.9

*Significant differences between groups using analysis of variance or chi-square tests

Of the 170 physicians who participated in the study, 99 were homeopaths and 71 conventional physicians. The 170 participants corresponded to 2.8% of all primary care providers in Switzerland listed by the Swiss medical association in 2002, and the participating homeopaths comprised 35% of all members of the SVHA.

We found significantly more female physicians in the HP group (31%) than in the CP group (13%). The levels of professional experience in both groups were similar; we found an average of 23 (CP) and 22 years (HP) since graduation. Homeopathic practices were significantly more often located in cities (57% versus 34%) than in rural areas (10% versus 17%). More conventional physicians were working in a single practice (72%) than homeopaths (51%). One third (34%) of the homeopathic doctors were working part-time, in contrast to 9% of their conventional peers. We found also differences in practice infrastructure: ECG, X-ray, ultrasound equipment, and laboratories were significantly more often present in CP practices[22].

Consultation times adjusted for gender and age of patients (LS-means) were significantly longer in the HP group, averaging 29 minutes, compared to 17 minutes in the CP group. HP physicians used exclusively homeopathic methods in 44% of all consultations and CP physicians used conventional procedures in 87% of all of their consultations (a detailed documentation of various other treatment combinations is given in table 2)

**Table 2:** Therapeutic procedures

		CP		HP	
		#	%	#	%
Specific therapeutic procedures	COM^a^	2693	87.0	575	19.5
	COM and homeopathy	1	0.0	318	10.8
	COM and other CAM^b^(without homeopathy)	18	0.6	51	1.7
	Homeopathy	8	0.3	1301	44.0
	Homeopathy and other CAM	-		196	6.6
	Others	52	1.7	276	9.3
	None	322	10.4	239	8.1

^a ^Conventional medicine

^b ^Complementary or alternative medicine (including anthroposophic medicine, TCM/acupuncture or neural therapy)

### Characteristics of patient population (Table 3)

**Table 3:** Demographic attributes and self rated health status of patients

		**CP**		**HP**	
		**#**	**%**	**#**	**%**
**Nr. of Patients **		1363	53.9	1702	47.5

**Age**^a^	Mean	53.93		47.47	

**Female Patients**^a^	Proportion	804	59.0	1276	75.0

**Patients with higher education**^b†^	Proportion	330	24.7	544	32.4

**General health**	excellent	63	4.7	62	3.7
	Very good	269	20.2	386	23.2
	good	697	52.4	884	53.0
	fair	254	19.1	298	17.9
	poor	46	3.5	37	2.2

**Chronic conditions**^b^	Proportion > 3 months	630	46.2	1018	59.8

Main health problems*	Proportion of subjective "severe" conditions	240	19.8	368	22.9

^a^Significant differences between groups using analysis of variance or chi-square test

^b^Significant differences between groups using logistic regression with age and gender as additional cofactors.

^† ^university or college degree

From the 6654 patients who completed the consultation questionnaire, 46% (n = 3065) returned the outcome questionnaire one month after the consultation. The proportion of responders was not significantly different between groups (HP 48%; CP 43%; p = 0.0503). Women responded significantly more often than men and chronic patients significantly more frequently than non-chronic patients.

Among the 3065 patients included in this study, 1702 patients consulted a HP with average age of 47; 75% were women. The patient group treated by CP consisted of 1363 persons with an average age of 54; a smaller percentage, 59%, were women. Patients of HP were significantly better educated (college or university degrees, 32%, versus. 25% of patients of CP), suffered significantly more often from chronic diseases (60% versus 46%) and claimed to have severe health problems significantly more frequently (23% versus 20%). Patients of HP judged their general health as "good," "very good," or "excellent" (together, 45%) more often than patients seeing a CP (34%).

The distribution of main diagnoses differed significantly between patient groups (see table 4 for details). The difference in the number of comorbid conditions between the groups was not significant (p = 0.18).

**Table 4:** Diagnoses and Comorbidities

**ICD-10 chapter**		**CP**	**HP**
		**%**	**%**
Diseases of the musculoskeletal system		17.5	16.8
Diseases of the respiratory system		9.9	10.6
Diseases of the circulatory system		17.7	5.9
Mental and behavioural disorders		8.2	6.1
Disease of the digestive system		6.3	6.2
Disease of the skin and subcutaneous tissue		3.4	5.1
Injury, poisoning		7.6	3.7
Diseases of the genitourinary system		3.1	6.3
Symptoms not elsewhere classified		3.5	7.5
Other diagnoses		22.8	31.8

**Comorbid conditions**	**None**	39.3	33.8
	**1**	29.6	34.3
	**> 1**	31.0	31.9

### Patient evaluations of treatment and of side effects (Table [Table T5])

**Table 5 T5:** Patient evaluations of treatment and side effects

		**CP**		**HP**	
		**#**	**%**	**#**	**%**
**Resolution of symptoms**	Proportion of "complete resolution"	358	27.58	347	20.90
**Fulfillment of treatment expectations**	Proportion of "complete fulfillment"	409	32.56	599	36.52
Treatment satisfaction*	Proportion of "completely satisfied"	549	43.40	871	52.66
Side effects*	Yes	192	15.38	155	9.26
	Mild	57		52	
	Moderate	121		100	
	Severe	14		3	
Other effects*	positive	208	17.11	650	40.55

*Significant differences between groups using logistic regression with age, gender, education and chronicity as additional cofactors

The proportion of patients reporting complete resolution of symptoms was non-significantly higher in the CP group than in the HP group (28% vs. 21%). However, patients of HP were significantly more often "completely satisfied" (53% vs. 43%) with their treatment, without significant differences in the fulfillment of their treatment related expectations (37% vs. 33%).

Side effects reported by patients were analysed for both CP and HP groups and for conventional and homeopathic therapies. A great majority of patients in CP and HP groups did not report any side effects. However, significant differences were observed between the groups. 15.4% of the CP patients reported side effects, compared to 9.3% of the HP group. This difference was also significant when we compared the side effects following pure conventional and pure homeopathic treatment (16.1% vs. 7.3%) (Fig. 1). Patients experiencing mild and moderate side effects were not significantly differently distributed between therapeutic groups (Table 5). Together they represented 92.7% in the CP and 98.1% in the HP group. Patients reporting severe side effects were significantly higher in CP group (7.3%) than in HP group (1.9%).

**Figure 2:**
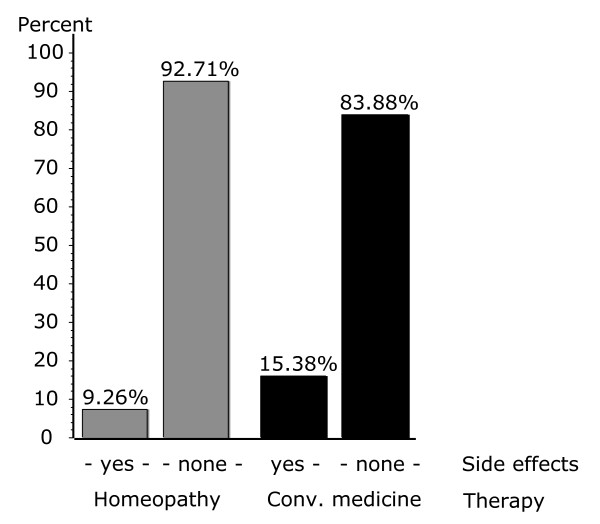
Side effects across treatment groups.

Finally, we analysed overall satisfaction rated as completely satisfied, mostly satisfied, mostly not satisfied, and not at all satisfied (fig. 2). The proportion of patients with complete satisfaction was significantly higher (52.6%) among patients treated by HP than by CP (43.4%), whereas more patients remained totally unsatisfied in the CP group.

### Europep questionnaire (Table 6)

**Table 6:** Patient satisfaction (Europep Questionnaire)

	**CP**	**HP**
**Questions/items % of answer excellent**	**%^e^**	**%^e^**

**Relation and communication**		
1. Making you feel you had time during consultation? *	61.7	75.4
2. Interest in your personal situation?*	60.3	73.7
3. Making it easy for you to tell him or her about your problem?*	62.9	71.6
4. Involving you in decisions about your medical care?	58.4	61.8
5. Listening to you? *	67.1	80.2
6. Keeping your records and data confidential? *	75.4	83.5

**Medical care**		
7. Quick relief of your symptoms?	27.6	25.3
8. Helping you to feel well so that you can perform your normal daily activities?	41.2	45.6
9. Thoroughness? *	56.5	70.0
10. Physical examination of you? *	52.6	47.3
11. Offering you services for preventing diseases (screening, health checks, immunizations)	48.7	46.0

**Information and support**		
12. Explaining the purpose of tests and treatments?	60.2	63.8
13. Telling you what you wanted to know about your symptoms and/or illness?	60.2	63.3
14. Helping you deal with emotional problems related to your health status?*	49.7	60.3
15. Helping you understand the importance of following his or her advice?	51.0	50.6

**Continuity and cooperation**		
16. Knowing what s/he had done or told you during earlier contacts? *	53.4	65.1
17. Preparing you for what to expect from specialist or hospital care?	55.7	56.3

**Facilities availability and accessibility**		
18. The helpfulness of the staff (other than the doctor)?	66.1	72.4
19. Getting an appointment to suit you?	1.2	1.8
20. Getting through to the practice on telephone? *	72.1	56.9
21. Being able to speak to the general practitioner on the telephone?	58.3	60.0
22. Waiting time in the waiting room? *	38.1	54.5
23. Providing quick services for urgent health problems?	71.6	71.0

*Significant differences between groups using logistic regression with age, gender, education and chronicity as additional cofactors

^e^proportion of ,,excellent" answers

For each of the first six questions of the Europep questionnaire, which are aimed at the relation and communication between patients and physicians, the proportion of the most favorable answers was higher in patients treated by homeopaths than by CP (significant differences for questions 1,2,3,5,6; see table 6). Answers regarding medical care were more varied. Thoroughness was rated significantly higher in homeopathic care. However, patients rated conventional care more highly for the physical examination during the consultation and offering services for preventing diseases. Regarding information and support, help with emotional problems was rated significantly better in patients of homeopaths, but for none of the other three questions were significant differences observed. For continuity and cooperation, only knowing what the physician did or said during earlier contacts was significantly rated as superior in patients treated by homeopaths. Finally, concerning facilities availability and accessibility, waiting time in the waiting room was significantly better rated in patients treated by homeopaths, whereas getting through to the practice by telephone was rated better by patients of CP. It appeared however, that obtaining suitable appointments was a common problem for all patients in the study.

## Discussion

The most interesting results of the present study are the striking difference in patient satisfaction and perception of side effects in conventional and homeopathic treatment. Our results confirm previous studies that show that patients of HP are more likely to be female, younger, to have a higher educational status, to suffer more often of chronic diseases, musculoskeletal problems, and mental disorders than patients of CP [4,10,11,23]

To the best of our knowledge this study is the first using the Europep questionnaire to investigate patient satisfaction comparing homeopathy and conventional care. The most significant differences concern doctor-patient relationship and communication. This is the first time, differences in communication patterns between CP and HP were reported. However, some limitations are to be taken into account:

1. The questionnaire used in the present study was not designed specifically for the assessment of homeopathy. Although it allowed determination of the frequency and severity of patients' side effects, it could not give further detailed information. For example, no distinctions were made between side effects related to the pharmacological properties of drugs, adverse events not necessarily caused by drugs, complications arising from adverse events following invasive interventions, homeopathic aggravations, and adverse drug reactions, which were all grouped as one entity: side effects. Due to these limitations, the influence of side effects and homeopathic aggravations on patient's satisfaction rate could not be determined precisely.

2. A single assessment one month after consultation does not sufficiently distinguish long-term from short-term effects. This was due to the overall limitations of the PEK study protocols and to the follow-up questionnaire after one month.

3. Different demographic attributes and higher educational level of the CAM patient population, and a potential overrepresentation of patients and physicians who were interested and motivated in the study may have positively biased the results towards homeopathy [23].

4. It may be argued that patients who were treated by HP physicians who used in specific cases exclusively CP procedures are misclassified with reference to the study groups. The rationale of maintaining this classification is given by the design of the overall project aimed at physicians and not at specific treatment procedures. Furthermore, specific properties of homoeopathic consultations may have been maintained by physicians even if only conventional procedures were applied.

5. Compliance in completing questionnaires may differ between CP and HP depending on their different commitment to this field of research and between satisfied and not satisfied patients.

6. Low participation of physicians was a problem in this study as physicians perceived the entire project as a government initated[24], which led to reservations to be involved. Furthermore, it must be assumed that the motivation among participating physicians was different, since HP physicians were under pressure to demonstrate effective methods–which was not the case for CP physicians. It can only be speculated that the motivation of CP physicians is more attributable to a general interest in primary care research. The generalisability of our results is therefore reduced to physicians with these distinct motivations. Nevertheless, a comparison of the sample population with the general population of all Swiss primary care providers indicated no difference with reference to geographic location of practices and gender of physicians; clinical data of the project including patient perceived health status with regard to other recent research in Swiss primary care showed also no difference[25,26]. Based on this additional information, we have no reason to consider our sample as well as our results as biased with regard to geographical distribution and gender of physicians or to health status of patients.

These findings reflect the fundamental differences between conventional and homeopathic medicine: in conventional care, a diagnosis is needed and specific problems are treated with specific procedures and medication. In homeopathy it is believed that the cause of all diseases is the disturbance of the person's life force, and all complaints are individual expressions of this[27]. Accordingly, homeopathic treatment is based on all reported or observed symptoms of the patient's body and personality. Indeed, the physician can be lost without the patient's co-operation, because collection of characteristic symptoms is the central issue of choosing the optimal homeopathic remedy [14]. This active role of the patient in both remedy-seeking process and healing process (taking responsibility for their health) may contribute to the positive assessment of the quality of communication and thoroughness by patients of HP [19,28]. These patient- and physician-related factors may also be the reason for greater thoroughness reported by patients of HP.

Two factors may be related to the high degree of satisfaction with homeopathic treatment despite of lower degree of symptom relief compared to the CP group: 1) physician's empathy manifested in detailed and holistic approach of homeopathic case-taking and consultation [15,29,30] and 2) existence of so-called "effectiveness gaps", chronic conditions where conventional therapies are either not available or not effective and which are then overrepresented among patients of HP [31].

The high percentage of complete fulfilment of treatment expectation among HP patients seems to be a contradiction to the low percentage of symptoms resolution in the same patient group (Table 5). A possible explanation is that both patients and physicians in homeopathy may give priority to a holistic and person-centered treatment approach aimed to increase self-healing capacities of patients [32,33]. These shared beliefs may also contribute to a better physician-patient communication and better patient satisfaction [34].

The other results of the Europep questionnaire showed less consistent answer patterns. However, with reference to emergency situations the question concerning "getting through to the practice on the telephone" was answered significantly more positively by patients of CP [35]. We suggest therefore that homeopaths should improve their accessibility by telephone. Building networks of homeopathic practices is one possibility [36].

In order to better understand 1) reasons for differences in the patient satisfaction between CP and HP and 2) the association between side effects and the overall satisfaction, we would need a further in-depth analysis of more detailed and different set of questionnaires than used in the present study.

## Conclusion

In a primary care setting, patient satisfaction is higher with homeopathic treatment compared to conventional treatment. Furthermore, certified homeopathic treatment is perceived as a low-risk therapy with less side effects than conventional treatment.

## Competing interests

The authors declare that they have no competing interests.

## Authors' contributions

FM participated in the development of the study and in data collection and wrote the final version of the manuscript. The first drafts of the manuscript were written by KJ (patient satisfaction) and KS (side-effects). KvA and AT reviewed and completed the manuscript and provided considerable input with reference to homeopathy and complementary medicine. AB was the principle investigator of the study, performed all statistical analyses and completed and reviewed the manuscript in this context.

## Pre-publication history

The pre-publication history for this paper can be accessed here:


